# Effect of age, sex, and hormonal state on tritiated thymidine uptake by rat pituitary.

**DOI:** 10.1038/bjc.1967.92

**Published:** 1967-12

**Authors:** W. A. Crane, R. S. Loomes


### EFFECT OF AGE, SEX, AND HORMONAL STATE ON TRITIATED THYMIDINE UPTAKE BY RAT PITUITARY

W. A. J. CRANE AND R. S. LOOMES

*From the Department of Pathology, University of Sheffield*

Received for publication July 21, 1967

PITUITARY tumours may arise spontaneously as certain strains of female rat become old, or they may be induced in rodents by a variety of experimental methods, chiefly involving hormonal imbalance. Less is known, however, of the factors which control the magnitude of cell division and growth in the pituitary, for mitotic figures are rare and conventionally the normal gland is regarded as a stable population of cells. A more accurate assessment of normal pituitary cell growth dynamics is obtained if tritiated ($^3$H) thymidine is used to localize by autoradiography nuclei in the DNA synthetic (S) phase of the nuclear cycle. A previous study with this technique showed that age and sex were important variables influencing $^3$H-thymidine labelling of the rat anterior pituitary, but these results were complicated by the fact that they were obtained from animals with various forms of experimental hypertension (Crane, Dutta and Ingle, 1965). Accordingly the experiments reported here were designed to study the influence of age, sex, the ovary and oestrus cycle, and the adrenal on the numbers of DNA-synthesizing nuclei in the rat pituitary, and to define more precisely the relationship of these factors to pituitary tumour induction.

### MATERIALS AND METHODS

Groups of male and female albino rats of an inbred strain were used. They were fed a commercial pellet diet *ad libitum* and given tap water, apart from adrenalectomized rats which drank 1% saline.

Bilateral adrenalectomy or oophorectomy was performed in 4 groups using a standard clean surgical technique. The rats were given a single injection of 5000 units of penicillin and 5 mg. of streptomycin after surgery and postoperative infection did not occur. ACTH (corticotrophin gel, Crookes) 10 units and hydrocortisone (hydrocortisone sodium succinate, Organon) 5 mg. were injected intramuscularly each day for 3 weeks in 2 further groups of male rats. In a further group of 30 female rats vaginal smears were examined daily for 3 weeks before killing the animals at various stages of the oestrus cycle.

Tritiated thymidine (thymidine-6-T nominal, Radiochemical Centre, Amersham) was injected intraperitoneally (0·7 $\mu$Ci/g. final body weight) at the end of each experiment. A constant 4 hour labelling time (10 a.m.—2 p.m.) was used throughout. At autopsy the relevant organs were fixed in 4% neutral formaldehyde after weighing. Autoradiographs were prepared from 5 $\mu$ paraffin sections with stripping film from Kodak AR10 plates (Crane and Dutta, 1963). The exposure time was 6 weeks at 4° C. in dry air. The image was developed with Kodak D 19b and sections were stained through the film with 1% aqueous neutral red.

Pituitary nuclei were counted with an oil-immersion objective and graticule eyepiece at $\times 1000$ magnification. The number of nuclei labelled with $^3$H-thymidine was expressed as a Label Index (number of radioactive nuclei per 100 pituitary nuclei). Approximately 6000 nuclei were counted in each anterior lobe using a standard scanning procedure which included the lateral and medial areas of the lobe, and peripheral and central fields in these areas. All the cells of the intermediate and posterior lobes in the particular section were counted (approximately 800 to 1000 nuclei).

### RESULTS

The results are in Tables I, II, and III. The distribution of DNA-synthesizing nuclei throughout the anterior lobe of the rat pituitary was not random in that counts of labelled nuclei were generally higher in the lateral wings than in the medial area of the lobe in most groups of rats. Further analysis of distribution, however, did not show any significant difference in counts taken from peripheral as compared with central fields within these areas. Labelled nuclei were seen occasionally in the endothelial cells of capillaries but these labels were specifically excluded from the counts to give as far as possible a population restricted to pituitary-cell nuclei.

### *Maturity*

Table I shows the effect of increasing age of male rats on the level of $^3$H-thymidine labelling of pituitary nuclei. The highest indices were given by the anterior

TABLE I.—*Frequency of $^3$H-Thymidine Labelled Nuclei in the Pituitary of Normal Male Rats at Various Ages*

| Body weight g. | Number of rats | Anterior lobe Lateral | Anterior lobe Medial | Intermediate lobe | Posterior lobe |
|---|---|---|---|---|---|
| 57 ±1·8 | 6 | 0·63 ±0·043 | 0·44 ±0·069 | 0·63 ±0·075 | 0·33 ±0·114 |
| 113 ±1·9 | 6 | 0·55 ±0·046 | 0·4 ±0·048 | 0·36 ±0·093 | 0·62 ±0·152 |
| 212 ±6 | 6 | 0·35 ±0·046 | 0·23 ±0·074 | 0·21 ±0·075 | 0·3 ±0·129 |
| 349 ±3·3 | 6 | 0·18 ±0·056 | 0·17 ±0·04 | 0·1 ±0·029 | 0·39 ±0·125 |

Figures are means ±S.E.

and intermediate lobes of young actively growing males of approximately 50 g. body weight. With older males of 100, 200, and 350 g. body weight respectively the number of DNA-synthesizing nuclei in the adenohypophysis diminished progressively at each weight interval and the lowest values were reached in the most mature males ($p < 0{\cdot}005$).

### *Sex and oestrus cycle*

Young female rats of approximately 50 g. body weight had the highest incidence of nuclear labelling in the adenohypophysis, the values for both the anterior ($p < 0{\cdot}0025$) and intermediate ($p < 0{\cdot}01$) lobes being significantly higher than the corresponding figures for males of comparable maturity (Tables I, II). When the pituitaries of older females of 200 g. body weight were studied it was clear that the stage of the oestrus cycle had an important influence on the frequency of $^3$H-thymidine labelling of anterior pituitary nuclei (Table II). The label

TABLE II.—*Influence of Age, Oestrus Cycle, Oophorectomy on the Frequency of $^3$H-Thymidine Labelled Nuclei in the Pituitary of Female Rats*

| Experiment | Number of rats | Final body weight g. | Anterior lobe Lateral | Anterior lobe Medial | Intermediate lobe | Posterior lobe |
|---|---|---|---|---|---|---|
| Normal immature | 6 | 48 ±1·4 | 1·28 ±0·169 | 1·17 ±0·125 | 1·13 ±0·192 | 1·09 ±0·103 |
| Pro-oestrus | 8 | 206 ±2·5 | 0·22 ±0·056 | 0·23 ±0·039 | 0·34 ±0·047 | 0·3 ±0·062 |
| Oestrus | 6 | 207 ±1·7 | 1·14 ±0·193 | 1·04 ±0·12 | 0·15 ±0·026 | 0·032 ±0·016 |
| Di-oestrus, day 1 | 8 | 205 ±1·9 | 0·21 ±0·039 | 0·22 ±0·053 | 0·35 ±0·072 | 0·28 ±0·065 |
| Di-oestrus, day 2 | 8 | 210 ±1·7 | 0·12 ±0·029 | 0·18 ±0·055 | 0·38 ±0·078 | 0·31 ±0·09 |
| Oophorectomy, 1 week | 6 | 206 ±3·6 | 0·31 ±0·059 | 0·31 ±0·06 | 0·34 ±0·14 | 0·31 ±0·064 |
| Oophorectomy, 2 weeks | 6 | 236 ±3·1 | 0·31 ±0·022 | 0·26 ±0·01 | 0·33 ±0·1 | 0·45 ±0·1 |

Figures are means ± S.E.

indices at pro-oestrus and di-oestrus, days 1 and 2, were of a low level corresponding to the values obtained from male rats of similar body weight. Rats in oestrus, however, showed a pronounced rise in the labelling frequency of anterior lobe nuclei ($p < 0{\cdot}0005$) to a level comparable to that observed in young actively growing females (50 g.). This increased frequency of DNA-synthesizing nuclei in the female anterior lobe at oestrus was not matched by a similar shift in the intermediate lobe. The label index of the latter in fact fell to the lowest value of any of the experimental groups.

Thymidine uptake was studied in 2 groups of 200 g. females at 1 and 2 weeks after oophorectomy. The results in both groups were similar. The label indices for the anterior and intermediate lobes were equivalent to or only marginally higher than the values obtained from females with intact ovaries at pro-oestrus and di-oestrus. The oestrus peak in the anterior lobe was abolished ($p < 0{\cdot}0025$) by oophorectomy and the labelling frequency in the intermediate lobe was not depressed.

*Adrenal factors*

Two groups of 200 g. male rats were given ACTH 10 units or hydrocortisone 5 mg. intramuscularly each day for 3 weeks. The frequency of $^3$H-thymidine labelling of the anterior lobe was slightly depressed in both groups when compared with males of similar body weight but there was no effect on the label index of intermediate lobe nuclei (Table III). The fall in the anterior lobe label index induced by ACTH ($p < 0{\cdot}025$) was more marked than the depression following hydrocortisone, the values for which did not reach levels required for statistical

TABLE III.—*Influence of Adrenalectomy, ACTH, Hydrocortisone on the Frequency of* $^{3}$*H-Thymidine Labelled Nuclei in the Pituitary of* 200 *g. Male Rats*

| Experiment | Number of rats | Final body weight g. | Anterior lobe Lateral | Anterior lobe Medial | Intermediate lobe | Posterior lobe |
|---|---|---|---|---|---|---|
| Normal | 6 | 212 ±6 | 0·35 ±0·046 | 0·23 ±0·074 | 0·21 ±0·075 | 0·3 ±0·129 |
| Adrenalectomy, 1 week | 6 | 203 ±1·3 | 1·02 ±0·078 | 0·92 ±0·08 | 0·55 ±0·119 | 0·34 ±0·125 |
| Adrenalectomy, 2 weeks | 5 | 224 ±8·2 | 0·92 ±0·095 | 0·65 ±0·058 | 0·47 ±0·058 | 0·68 ±0·112 |
| ACTH, 10 units daily for 3 weeks | 6 | 250 ±5·6 | 0·18 ±0·012 | 0·12 ±0·025 | 0·2 ±0·034 | 0·14 ±0·034 |
| Hydrocortisone, 5 mg. daily for 3 weeks | 5 | 252 ±4·3 | 0·29 ±0·028 | 0·14 ±0·025 | 0·23 ±0·05 | — |

Figures are means ± S.E.

significance. Three of the hydrocortisone-treated rats showed mild glycosuria in the last week of the experiment. Previous observations of male rats given cortisone 5 mg. daily for a 6 week period showed a profound depression of anterior lobe labelling (label index = 0·038 ± 0·02 S.E.) and in all the animals so treated a persistent and more severe glycosuria had resulted (Crane, *et al.*, 1965).

Bilateral adrenalectomy was performed in 2 groups of 200 g. male rats and the frequency of $^{3}$H-thymidine labelling of the pituitary was determined 1 and 2 weeks after operation. The label indices of the anterior and intermediate lobes were both significantly increased above the values for male rats of similar body weight with intact adrenals ($p < 0{\cdot}0005$). The magnitude of the increase in the anterior lobe following adrenalectomy approached the levels obtained by the normal female rat at oestrus (Tables II, III) but whereas intermediate lobe labelling was depressed at oestrus, removal of the adrenals increased the frequency of DNA-synthesizing nuclei in this area in the male.

*Posterior lobe*

Alterations in the label index of the posterior lobe were less common and less clearly related to the experimental design. The highest index was observed in young 50 g. females and the lowest in 200 g. females at oestrus.

### DISCUSSION

Although the frequency of mitotic division in the pituitary is low, the $^{3}$H-thymidine autoradiographic technique is sufficiently sensitive to permit meaningful assessments of pituitary cell proliferative activity. This is due partly to the ease with which the labelled nuclei can be identified and counted, as compared with mitoses, but relates also to the duration of the DNA-synthetic (S) phase of the nuclear cycle which is approximately 8 to 10 times longer than the time occupied by nuclear division (Patt and Quastler, 1963). In consequence a low label index of 0·18 for mature 350 g. male rats, indicating 1·8 nuclei per 1000 in the S-phase, can be reliably measured and distinguished from the increased proliferative activity in comparable areas of the anterior pituitary of young 50 g. males (6·3 labelled nuclei per 1000; Table I).

As normal male rats age the numbers of DNA-synthesizing nuclei in the anterior and intermediate lobes progressively decline. Each body-weight group forms a homogeneous population, with little internal variation (Table I). Presumably this reflects the constant 4 hour labelling interval we have used, as well as the uniform time of killing the animals (2 p.m.), so excluding variations due to circadian rhythms which are known to affect the nuclear cycle of other tissues (Pilgrim, Erb and Maurer, 1963).

The anterior and intermediate pituitary lobes of young 50 g. female rats have a higher proliferative activity than males of similar body weight. There is also a fall in labelling frequency as females age. Sexually mature females, however, do not form a homogeneous population with regard to $^3$H-thymidine labelling of the pituitary (Crane *et al.*, 1965). This is due to the rhythmic effect on the anterior pituitary of the oestrus cycle which induces in relation to each oestrus phase a burst of proliferative activity, as shown by the increased number of anterior pituitary nuclei labelled with $^3$H-thymidine. Our results in this respect agree with those of Hunt and Hunt (1967) who with a different counting technique recently reported an increase at oestrus in the labelling frequency per unit area of the adenohypophysis of Long-Evans strain female rats. Presumably this cyclical proliferative activity is matched by a corresponding loss of cells, possibly by some form of cellular degeneration, so to maintain the gland at a constant size through the sexual life of the female. We have further shown that this oestrus peak is prevented by oophorectomy. There is evidence to indicate that oestrogenic steroids have an important influence on cell growth dynamics in the pituitary. Some years ago Hunt (1947) showed that injections of oestrogen stimulated mitotic activity in the rat adenohypophysis, and it is well known that the implantation into rats of oestrogenic substances, such as diethylstilboestrol, will induce pituitary tumours which eventually become autonomous (Clifton and Furth, 1961).

Adrenalectomy significantly increases proliferative activity in the anterior pituitary whereas ACTH or hydrocortisone administration have the opposite effect. Although changes in $^3$H-thymidine uptake are not necessarily equated with corresponding alterations in hormone biosynthesis, our findings parallel the known inter-relationships of the anterior pituitary and the adrenal cortex. Hydrocortisone reduces the number of cells in DNA synthesis in various normal tissues (Young and Crane, 1964) and in the kidney undergoing compensatory growth (Bury, Crane and Dutta, 1965). An exception to this depressant effect is provided by the pancreatic islet-cells for increased numbers of DNA-synthesizing nuclei are found in the hyperplastic islets of rats with steroid diabetes resulting from cortisone overdosage (Crane and Dutta, 1964).

Where the $^3$H-thymidine labelling rate on autoradiography is high, identifiable mitoses would be expected in the corresponding routine histological preparations of anterior pituitary. We have seen small numbers of mitotic figures in the anterior pituitary of young 50 g. rats of both sexes, females at oestrus, and adrenalectomized males. The type of autoradiographic technique we have used so far does not permit identification of the specific pituitary cells engaged in DNA synthesis.

SUMMARY

The numbers of DNA-synthesizing nuclei in the rat pituitary were measured by autoradiography after flash-labelling with $^3$H-thymidine.

The frequency of $^3$H-thymidine labelled nuclei in the anterior and intermediate lobes of male rats fell progressively with age.

All lobes of the pituitary of young immature female rats contained more DNA-synthesizing nuclei than the corresponding pituitary areas of young males. At oestrus young mature female rats showed a marked rise in the $^3$H-thymidine labelling frequency in the anterior pituitary lobe as compared with the low levels at the other stages of the oestrus cycle. This oestrus peak was abolished by oophorectomy.

Adrenalectomy in male rats increased the numbers of DNA-synthesizing nuclei in the anterior and intermediate pituitary lobes, whereas the administration of ACTH or hydrocortisone depressed the numbers of $^3$H-thymidine labelled nuclei.

This investigation was supported by a grant from the British Empire Cancer Campaign for Research. The assistance of Miss Barbara Codling in preparing the autoradiographs is gratefully acknowledged.

REFERENCES

BURY, H. P. R., CRANE, W. A. J. AND DUTTA, L. P.—(1965) *Br. J. Urol.*, **37**, 201.

CLIFTON, K. H. AND FURTH, J.—(1961) *Cancer Res.*, **21**, 913.

CRANE, W. A. J. AND DUTTA, L. P.—(1963) *J. Path. Bact.*, **86**, 83.—(1964) *J. Endocr.*, **28**, 341.

CRANE, W. A. J., DUTTA, L. P. AND INGLE, D. J.—(1965) *Proc. Soc. exp. Biol. Med.*, **119**, 167.

HUNT, T. E.—(1947) *Anat. Rec.*, **97**, 127.

HUNT, T. E. AND HUNT, ELEANOR A.—(1967) *Anat. Rec.*, **156**, 361.

PATT, H. M. AND QUASTLER, H.—(1963) *Physiol. Rev.*, **43**, 357.

PILGRIM, C., ERB, W. AND MAURER, W.—(1963) *Nature, Lond.*, **199**, 863.

YOUNG, M. H. AND CRANE, W. A. J.—(1964) *Ann. rheum. Dis.*, **23**, 163.


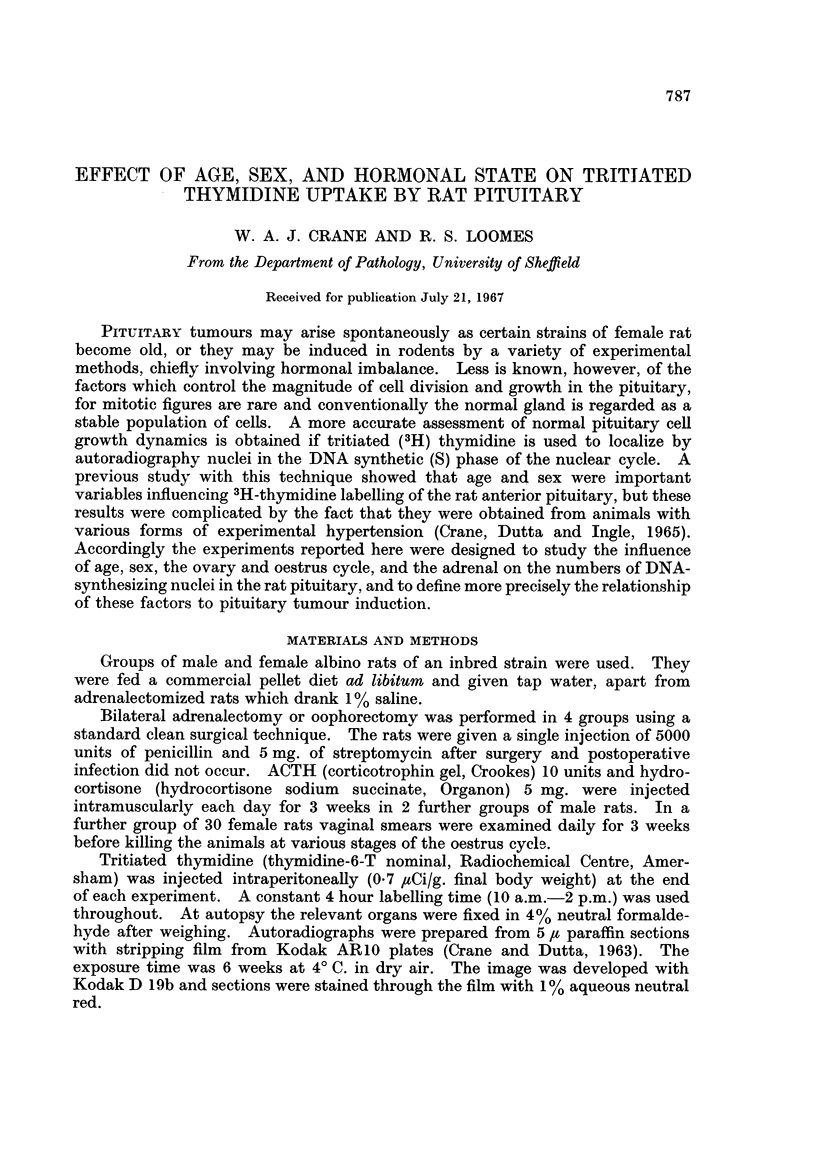

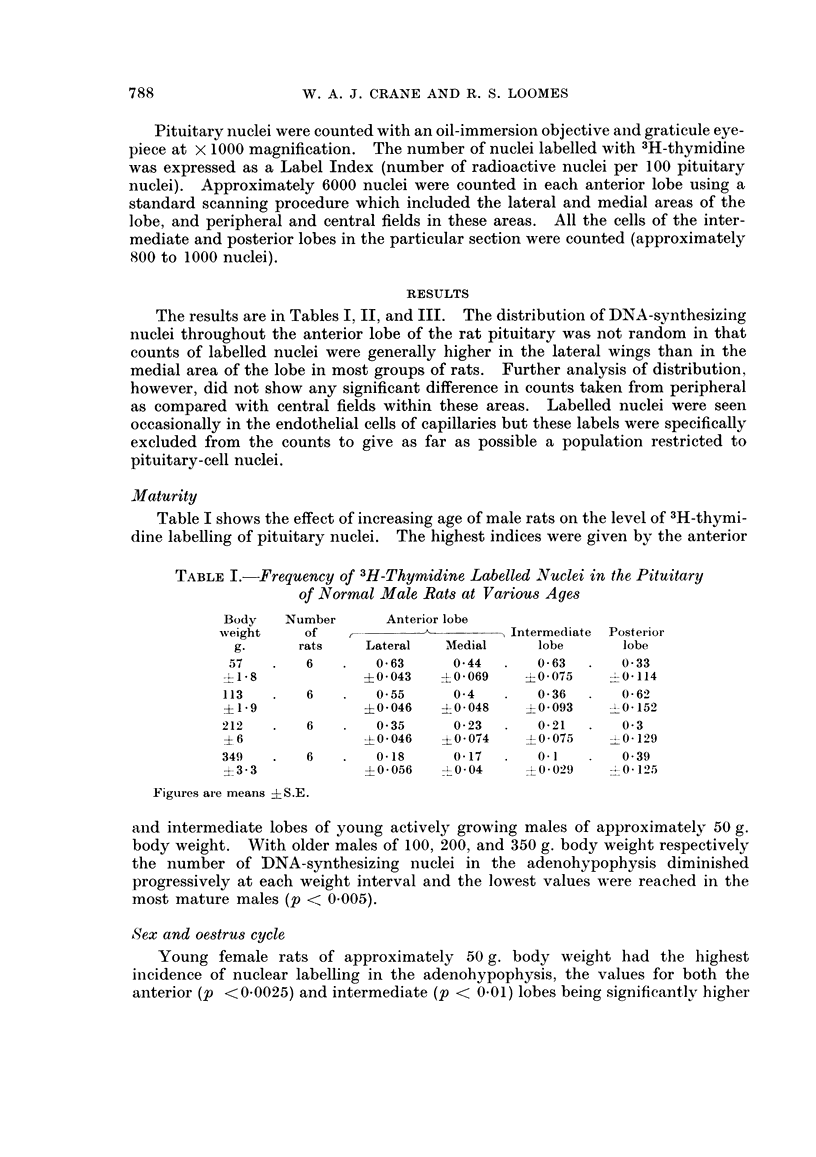

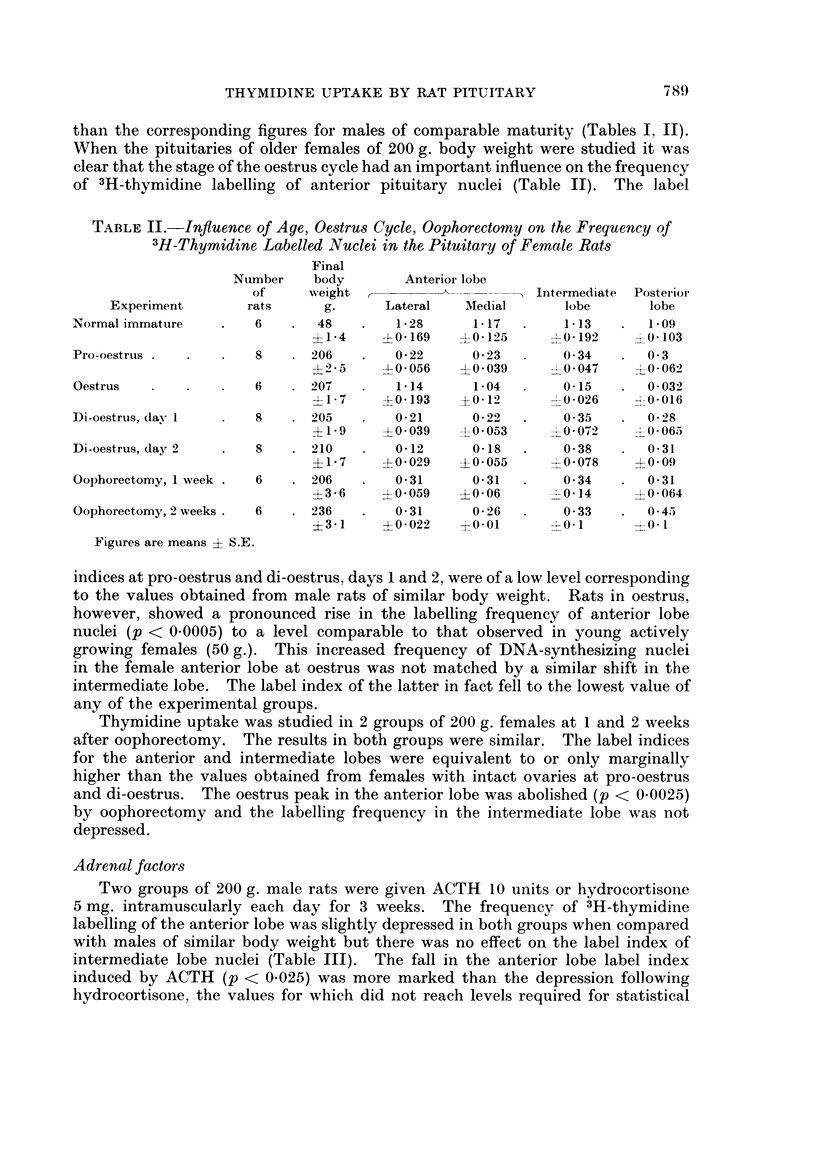

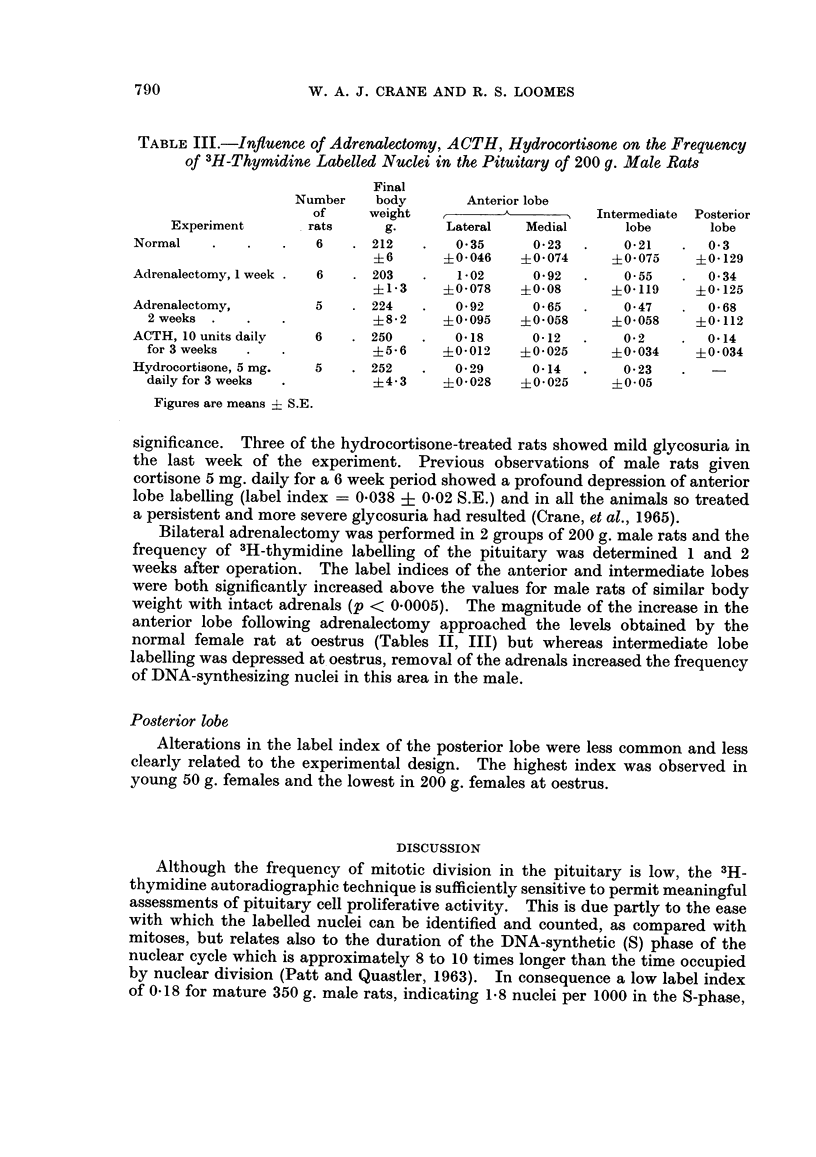

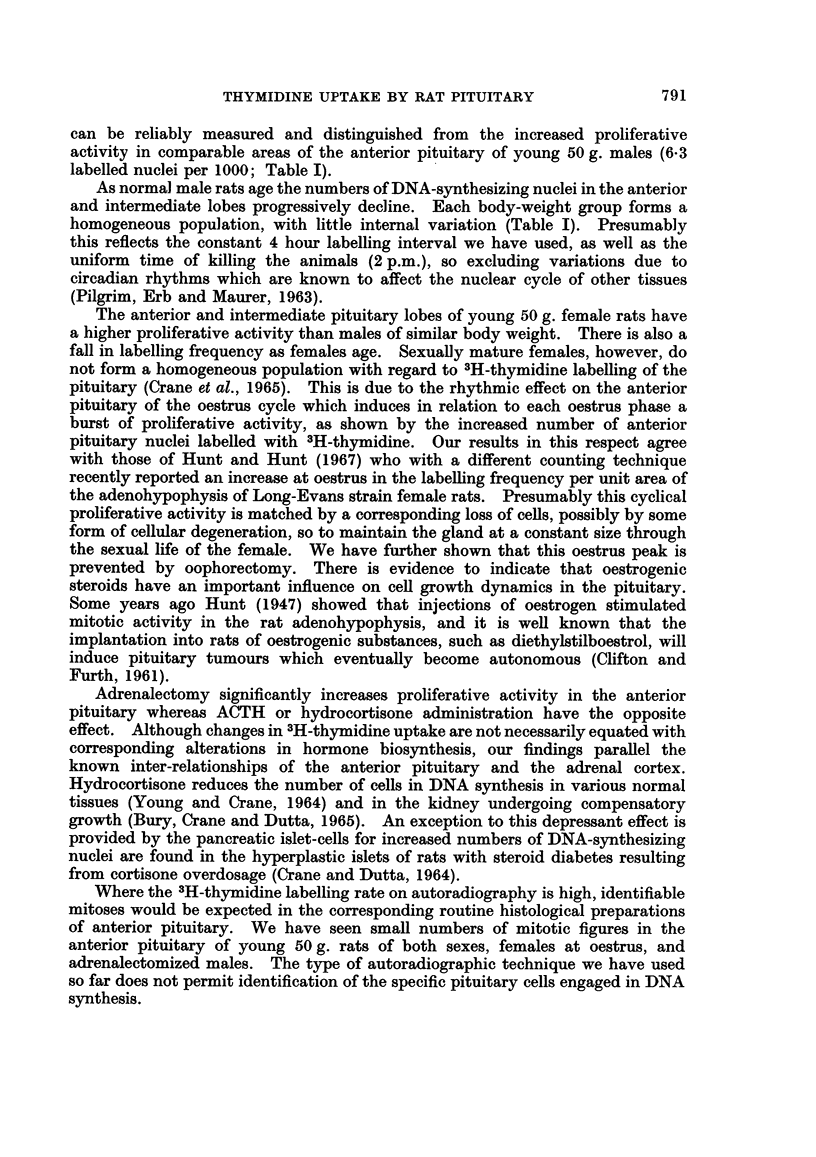

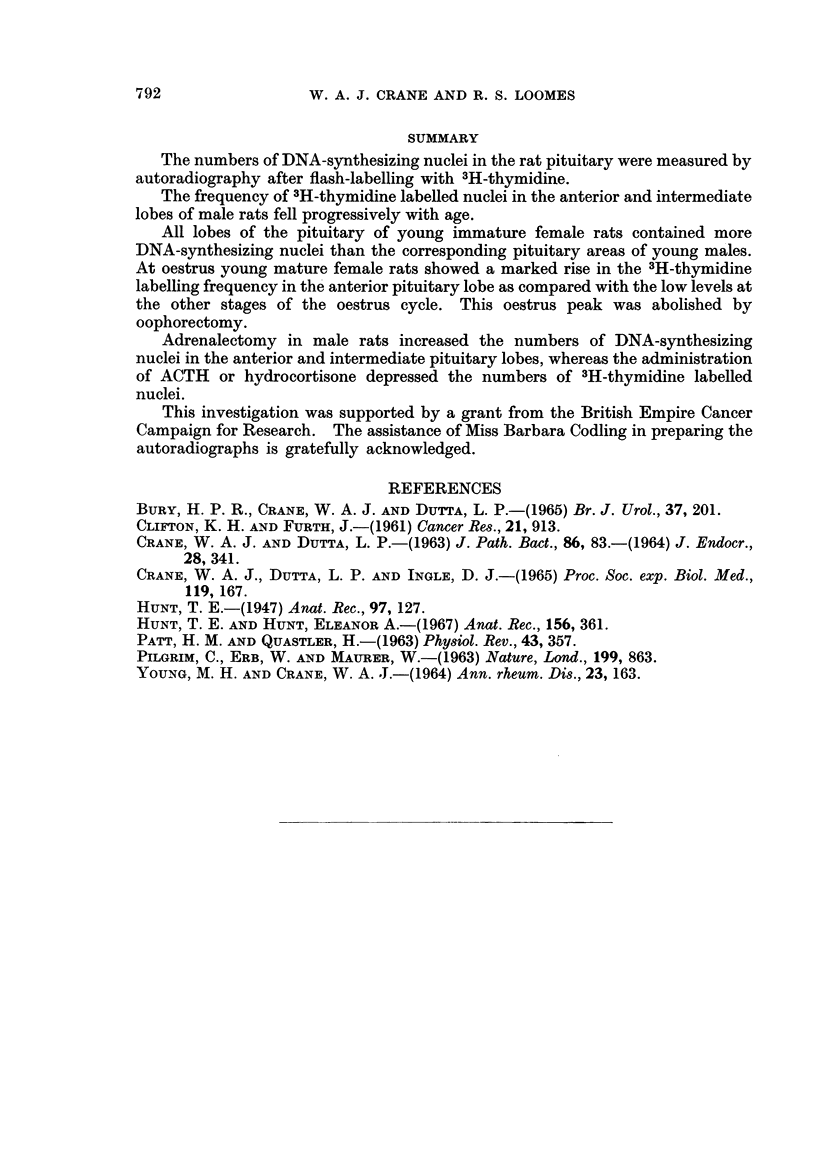


## References

[OCR_00338] BURY H. P., CRANE W. A., DUTTA L. P. (1965). CELL PROLIFERATION IN COMPENSATORY RENAL GROWTH.. Br J Urol.

[OCR_00345] CRANE W. A., DUTTA L. P., INGLE D. J. (1965). CELL PROLIFERATION IN THE RAT PITUITARY.. Proc Soc Exp Biol Med.

[OCR_00341] CRANE W. A., DUTTA L. P. (1964). THE INFLUENCE OF AGE AND HORMONAL STATUS ON THE UPTAKE OF TRITIATED THYMIDINE BY RAT PANCREAS.. J Endocrinol.

[OCR_00352] PATT H. M., QUASTLER H. (1963). Radiation effects on cell renewal and related systems.. Physiol Rev.

[OCR_00354] PILGRIM C., ERB W., MAURER W. (1963). DIURNAL FLUCTUATIONS IN THE NUMBERS OF DNA SYNTHESIZING NUCLEI IN VARIOUS MOUSE TISSUES.. Nature.

[OCR_00355] YOUNG M. H., CRANE W. A. (1964). EFFECT OF HYDROCORTISONE ON THE UTILIZATION OF TRITIATED THYMIDINE FOR SKELETAL GROWTH IN THE RAT.. Ann Rheum Dis.

